# Low serum total nitrite and nitrate levels in severe leptospirosis

**DOI:** 10.1186/1471-2334-13-206

**Published:** 2013-05-06

**Authors:** Thilini Kalugalage, Chaturaka Rodrigo, Thamal Vithanage, Pranitha Somaratne, H Janaka De Silva, Shiroma Handunnetti, Senaka Rajapakse

**Affiliations:** 1 Institute of Biochemistry, Molecular Biology and Biotechnology, University of Colombo, Colombo, Sri Lanka; 2 Department of Clinical Medicine, Faculty of Medicine, University of Colombo, Colombo, Sri Lanka; 3 Medical Research Institute, Colombo, Sri Lanka; 4 Department of Medicine, Faculty of Medicine, University of Kelaniya, Kelaniya, Sri Lanka

**Keywords:** Leptospirosis, Nitric oxide, Nitrate, Nitrite, Severity, Creatinine

## Abstract

**Background:**

The relationship between inducible nitric oxide synthatase activity and disease severity in leptospirosis is unclear. Nitric oxide is converted to nitrites and nitrates, thus nitrite and nitrate levels (NO^x^) in serum are considered surrogate markers for nitric oxide. NO_x_ are excreted through the kidneys, and elimination is diminished in renal impairment. We assessed the correlation of NO_x_ with disease severity in patients with leptospirosis, compared with healthy controls and non-leptospirosis fever patients.

**Methods:**

All patients admitted over a two-month period to the National Hospital, Colombo, Sri Lanka with a clinical picture suggestive of leptospirosis were included. Leptospirosis was confirmed by the microscopic agglutination test (titre≥400). Severe leptospirosis was defined by the presence of two or more of the following criteria: jaundice (bilirubin> 51.3 μmol/l), oliguria (urine output < 400 ml/day), serum creatinine> 133 μmol/l or blood urea > 25.5 mmol/l, or the presence of organ dysfunction. Non-leptospirosis fever patients and healthy volunteers were used as control groups. NO_x_ levels were measured using a modified Griess reaction.

**Results:**

Forty patients were confirmed as having leptospirosis and 26 of them had severe disease. NO_x_ levels were significantly higher in confirmed leptospirosis patients compared to healthy controls, MAT equivocal patients and non-leptospirosis fever patients (p<0.001). NO_x_ concentrations were also significantly higher in patients with severe compared to mild leptospirosis (p<0.001). Once NO_x_ levels were corrected for renal function, by using the ratio NO_x_/creatinine, NO_x_ levels were actually significantly lower in patients with severe disease compared to other patients, and values were similar to those of healthy controls.

**Conclusions:**

We postulate that high NOx levels may be protective against severe leptospirosis, and that finding low NOx levels (when corrected for renal function) in patients with leptospirosis may predict the development of severe disease and organ dysfunction.

## Background

Leptospirosis is a zoonotic illness that has a high morbidity and mortality in the tropics [1]. It is caused by a spirochaete of the genus *Leptospira,* which is now found to have at least nine pathogenic species and over 250 serovars. The global burden of leptospirosis is difficult to quantify due to under-reporting and difficulties in establishing a serological diagnosis. However, it is estimated that in endemic areas (localized geographical areas in Central America, Indian subcontinent, Oceania, and the Caribbean), the incidence of leptospirosis can be as high as 25 clinical infections per 100,000 of population per year (in contrast to 1 per 100,000 per year in non-endemic areas) [2].

The majority of infections are asymptomatic or may pass off as a flu like illness. However, severe leptospirosis can be fatal. Severe leptospirosis is associated with adult respiratory distress syndrome (ARDS), pulmonary haemorrhage, acute kidney injury, liver impairment, and multi-organ dysfunction syndrome (MODS) [3,4]. The case fatality in severe leptospirosis (Weil’s disease) can be as high as 40% [5].

Predictors of disease severity can be useful to the clinician for anticipating complications. Factors predicting mortality in severe leptospirosis as reported in various studies worldwide have been reviewed by Rajapakse et al. [6] under the categories of predisposition, insult, response and organ dysfunction (similar to the PIRO model used to predict mortality in severe sepsis). Although there was insufficient data to develop a scoring system for mortality prediction, it was noted in this review that serum markers of acute inflammation (tumour necrosis factor-α, interleukin-1, interleukin-6) have not been adequately assessed as prognostic markers. These pro-inflammatory cytokines lead to an increase in the activity of inducible nitric oxide synthatase (iNOS) to synthesize nitric oxide (NO) which is toxic to the bacterium. The role of iNOS and NO production in inflammation has not been clearly determined in leptospirosis; in fact its significance in severe sepsis [7] and other infections such as malaria [8-11] is subject to much debate [12,13]. While its primary role is to combat infection, NO levels have been shown to be elevated in severe leptospirosis [14], leading to the postulate that high NO levels may be involved in the pathogenesis of organ dysfunction in leptospirosis. On the other hand, increased iNOS activity may actually protect against organ dysfunction.

NO is an extremely volatile compound that is difficult to measure in serum. It is quickly converted to nitrite (NO_2_^ˉ^) and nitrate (NO_3_^ˉ^) [15]. It is estimated that more than 95% of nitrite in whole blood gets converted to nitrate within one hour [16]. Thus, the total blood levels of nitrite and nitrate (NO_x_) could be considered to be a surrogate marker of serum NO levels. NO_x_ levels in blood are affected by the amount of ingested nitrates; to control for this, measurements should ideally be made after an overnight fast [17]. Furthermore, NO_x_ is excreted renally, thus NO_x_ clearance is reduced in the presence of renal impairment [18]. To correct for this, the use of the ratio of serum NO_x_/creatinine has been suggested to be a more accurate marker of iNOS activity than crude NO_x_ levels, and was used in the study by Anstey et al. [17] to correct NO_x_ levels for renal function in patients with malaria. Although other factors could influence creatinine levels, and therefore this correction factor too, creatinine levels are the standard index used for assessment of renal dysfunction in acute kidney injury [19]. Therefore the formula NO_x_/creatinine is currently the only practical formula available to correct NO_x_ levels for renal function.

We previously demonstrated through a preliminary study that serum nitrite levels are elevated in patients with acute leptospirosis compared to healthy controls [20]. However, the sample size was inadequate to determine a correlation with disease severity. The aim of this study was to determine the relationship between NO_x_ (i.e., total nitrite and nitrate) levels in the blood (as a marker of iNOS activity) and disease severity in leptospirosis. If such a correlation exists, NO_x_ could potentially be useful as a prognostic marker.

## Methods

### Objectives

The objectives of our study were to a) determine serum NO_x_ (nitrate and nitrite) levels in patients with confirmed leptospirosis, b) compare serum NO_x_ levels in leptospirosis patients with mild and severe disease, healthy controls and non-leptospirosis fever patients (NLFs), and c) seek a correlation between serum NO_x_ levels and disease severity after correcting for impaired renal clearance.

### Participants

Patients suspected to have leptospirosis were selected from the National Hospital of Sri Lanka (NHSL). The NHSL is the premier tertiary care center in Sri Lanka, with a bed strength of over 3600. It is one of the few state sector centers with facilities for haemodialysis in the country, and most patients with acute kidney injury are transferred to NHSL for further management. It is also the major hospital that covers the heavily populated Western Province which is an endemic area for leptospirosis [21]. The annual incidence of leptospirosis in the Western Province for the year 2011 was 22 per 100,000 population.

All patients with a febrile illness who were clinically suspected of having leptospirosis admitted to medical wards in NHSL during a two-month period from 23rd June to 27th August 2010 were included in to the study, after obtaining informed consent. The clinical criteria to define a probable case of leptospirosis were adopted from the World Health Organization (WHO) surveillance criteria [22]. Microscopic Agglutination Titre (MAT) is the most widely used confirmatory test for leptospirosis, although the duration that MAT remains positive after infection is not clearly known [23]. Nonetheless a MAT titre of > 400 is generally considered to indicate acute infection even in areas of high endemicity, in the setting of a clinical diagnosis of leptospirosis. Based on MAT results, patients were retrospectively categorized as confirmed leptospirosis (MAT titre≥400, MAT equivocal (MAT titre 100 and 200), and non-leptospirosis fever (MAT negative). Healthy volunteers (MAT negative) were selected as controls.

Serial haematological and serum biochemical measurements of patients were made during the illness, and included leukocyte and platelet counts, blood culture, erythrocyte sedimentation rate (ESR), serum potassium, serum sodium, aspartate aminotransferase, alanine aminotransferase, creatinine, blood urea and indirect, direct and total bilirubin levels.

Patients with severe leptospirosis were defined as those presenting with acute fever and clinical symptoms compatible with leptospirosis (confirmed serologically by a positive MAT result) with two or more of the following criteria: jaundice (bilirubin> 51.3 μmol/l), oliguria (urine output < 400 ml/day), serum creatinine> 133 μmol/l or blood urea > 25.5 mmol/l [14], or the presence of acute organ dysfunction.

### Determination of serum NO_X_ levels

Serum NO_x_ levels were determined in all patients who were recruited in to the study. NO_x_ levels were measured in blood obtained early morning. Total NO_x_ levels were used as a surrogate marker for serum nitric oxide levels [24].

The blood samples collected were centrifuged, and separated sera were stored at −20°C. During analysis, the serum samples were first thawed, then deproteinized by adding zinc sulfate. Deproteinization is a necessary step in the measurement of serum nitrite concentrations [25]. Ten microlitres of 1.5 g/mL zinc sulphate solution was added to 1mL of serum, vortexed for 1 minute, and centrifuged at 10,000 *g* for 10 minutes at room temperature (RT=25°C, i.e., the controlled temperature in the laboratory). The supernatant was pipetted out and centrifuged again at 10,000 *g* for 10 minutes. The clear serum (100 μL) was applied in duplicate to a 96-well ELISA plate, 100 μL of vanadium (III) chloride (8 mg/mL) was added to each well (for reduction of nitrate to nitrite) followed by the addition of 100 μL of Griess reagent (equal mixture of 1% sulphanilamide in 5% phosphoric acid and 0.1% N-(1-naphthyl) ethylenediamine hydrochloride in distilled water). The plates were incubated for 30 minutes at RT and the optical density was measured at 540 nm using the ELISA reader (Bio-Tek Instruments INC, USA). A two-fold dilution series (0.193 - 100 μM) of NaNO_2_ was prepared from 100 μM NaNO_2_ solution using distilled water. Each dilution (100 μL) was mixed with an equal volume of Griess reagent, and the optical density (OD) was measured at 540 nm. A standard curve was plotted against optical density and NaNO_2_ concentration. Intra-assay coefficient of variability (CV) was 6.55% and inter-assay CV was 8.62%, which indicated good precision and repeatability.

### Ethics

Ethics approval was obtained from the Ethics Review Committee of the Faculty of Medicine, University of Colombo and the Ethics Review Committee of the NHSL. Informed written consent was obtained from all patients and healthy controls prior to recruitment to the study.

### Statistical methods

Statistical analysis was performed using SPSS® version 17.0. Results were expressed as mean ± SD. Data were analyzed by applying a one-way ANOVA with the Bonferonni/Dunn post-hoc correction for multiple comparisons. Multivariate regression analysis was performed to determine the relationship between NO_x_ and other biochemical markers of severity. Statistical significance was defined as p < 0.05.

## Results

### Patient categorization

On the basis of clinical features, 85 patients were recruited to the study with probable leptospirosis. Of these, 40 were confirmed as leptospirosis with MAT (titre of ≥400). There were also 27 patients who had equivocal MAT titers and 18 with non-leptospirosis fever (MAT negative). Twenty three MAT negative healthy individuals were also recruited as controls. The mean ± SD of the duration between onset of symptoms and obtaining serum samples in the confirmed leptospirosis patients was 10.6 ± 3.9 days. The baseline characteristics of these groups are shown in Table 1.

**Table 1:** Comparison of age and gender among the study groups

	**Confirmed leptospirosis patients**	**MAT equivocal patients**	**Non-leptospirosis fever controls**
Number of patients	40	27	18
MAT titre	≥ 400	100-200	0
Mean age ± SD (years)	39.7 ± 14.6	38.4 ± 15.2	37.8 ± 13.8
Gender (Male:Female)	19 : 1	26 : 1	8 : 1

Of the 40 patients with confirmed leptospirosis, 26 were categorized as having severe disease according to the criteria mentioned above. One patient with severe disease died and all others survived. The laboratory investigations for each category of patients and for non-leptospirosis fever patients are summarized in Table 2.

**Table 2:** Laboratory parameters of mild and sever leptospirosis patients, MAT equivocal patients and non-leptospirosis fever controls (univariate analysis)

**Laboratory parameter†**	**Patients with severe leptospirosis**	**Patients with mild leptospirosis**	**MAT equivocal group**	**Non-leptospirosis fever patients**
WBC-highest (cells/mm^3^)	15461 ± 9625	11418 ± 5071	12170 ± 3637	11650 ± 2703
Platelets-lowest (cells/mm^3^)	114714 ± 91166	142154 ± 72178	NA	NA
ESR-highest (mm/hour)	59.8 ± 42.7	45.6 ± 41.1	47.6 ± 40.8	45.3 ± 23.9
Bilirubin-total (μmol/l)	159.0 ± 140.2*	49.7 ± 37.2	52.1 ± 40.6	61.29 ± 69.5
AST (U/L)	138.6 ± 90.0	97.5 ± 75.3	140.9 ± 141.7	89.8 ± 68.7
ALT (U/L)	105.4 ± 58.0	94.6 ± 63.3	89.7 ± 59.9	62.4 ± 27.7
Blood urea (mmol/l)	16.0 ± 8.3*	5.7 ± 5.4	6.7 ± 3.9	6.7 ± 4.6
Serum creatinine (μmol/l)	520.4 ± 200.4*	113.3 ± 15.6	153.6 ± 142.9	140.1 ± 82.1

† mean ± SD; NA – not available; *differed at a statistically significant level (p<0.001) from patients with mild disease and MAT equivocal patients.

### Comparison of uncorrected serum NO_X_ levels

**Figure 1:**
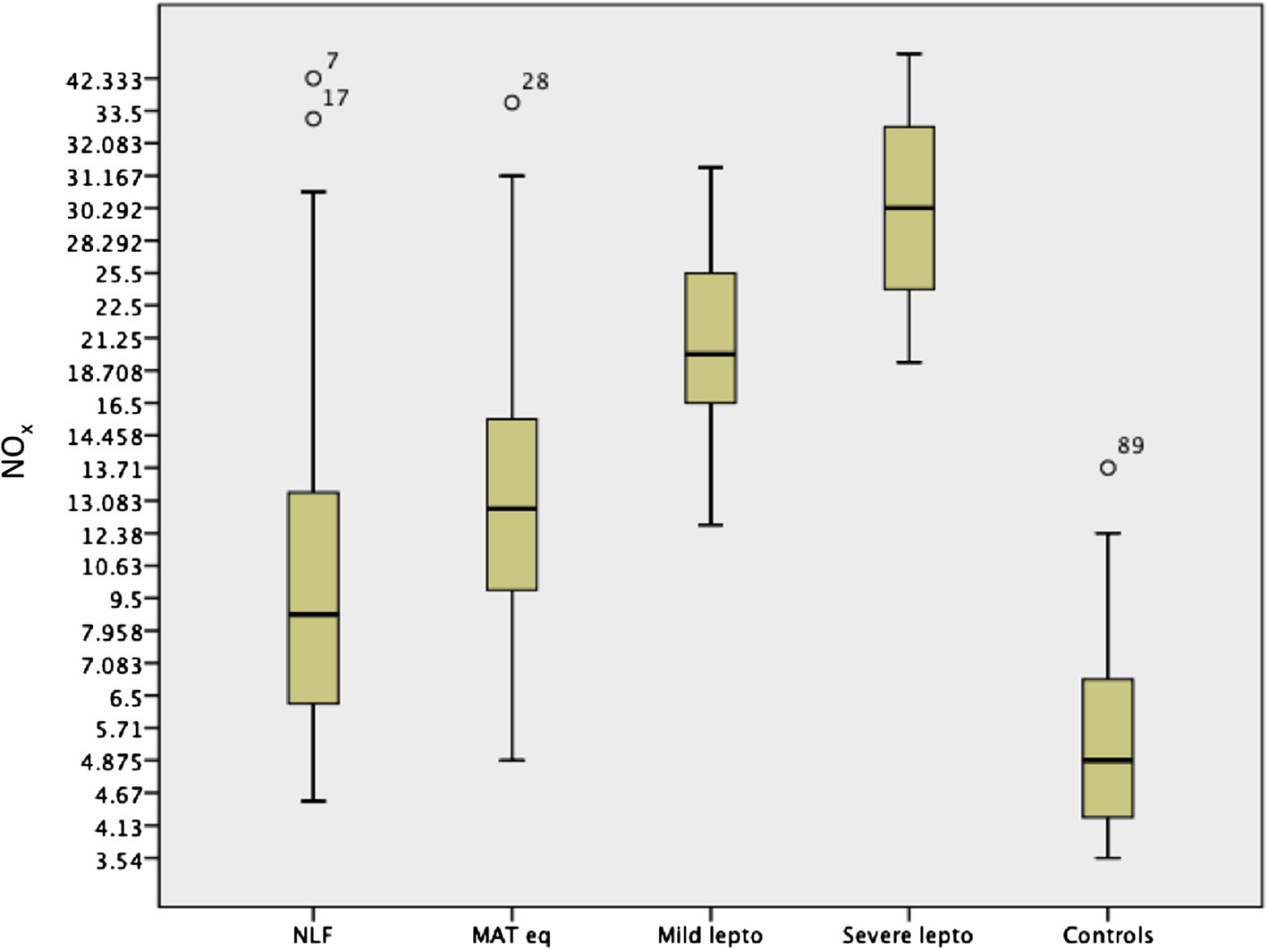
**Serum NO**_**x **_**levels in different patient groups and healthy controls.** NLF: Non-leptospirosis fever; MAT Eq: MAT equivocal patients. lepto: leptospirosis.

We first compared NO_x_ levels of confirmed leptospirosis patients (mild and severe) MAT equivocal patients, NLF patients, and healthy controls (Table 3 and Figure 1, Table 4). Significantly higher NO_x_ levels were observed in confirmed leptospirosis patients when compared against healthy controls, MAT equivocal patients and non-leptospirosis fever patients (p<0.001). NO_x_ concentrations in patients with severe leptospirosis were also significantly higher than in those with mild leptospirosis (p=0.003). There was no significant difference in values between MAT equivalent patients and non-leptospirosis fever patients. Notably, many of these differences were not observed when the serum nitrite levels alone were considered.

**Table 3:** ANOVA post-hoc comparison using Bonferonni correction comparing uncorrected NOx in the different groups

**(I) Category**	**(J) Category**	**Mean difference (I-J)**	**Std. error**	**Sig.**
NLF	MAT eq	-.509167	2.661898	1.000
	Mild lepto	−7.622690	3.117296	.162
	Severe lepto	−18.547372^*^	2.682296	.000
	Controls	7.444000	2.727634	.075
MAT equivocal	NLF	.509167	2.661898	1.000
	Mild lepto	−7.113524	2.881040	.152
	Severe lepto	−18.038205^*^	2.403658	.000
	Controls	7.953167^*^	2.454149	.016
Mild leptospirosis	NLF	7.622690	3.117296	.162
	MAT eq	7.113524	2.881040	.152
	Severe lepto	−10.924681^*^	2.899897	.003
	Controls	15.066690^*^	2.941883	.000
Severe leptospirosis	NLF	18.547372^*^	2.682296	.000
	MAT eq	18.038205^*^	2.403658	.000
	Mild lepto	10.924681^*^	2.899897	.003
	Controls	25.991372^*^	2.476259	.000
Healthy Controls	NLF	−7.444000	2.727634	.075
	MAT eq	−7.953167^*^	2.454149	.016
	Mild lepto	−15.066690^*^	2.941883	.000
	Severe lepto	−25.991372^*^	2.476259	.000

NLF: Non-leptospirosis fever, MAT eq: MAT equivocal patients, lepto: leptospirosis. Sig: significance. * denotes statistically significant differences.

**Table 4:** **NO**_**X **_**values in the different categories**

**Category**	**N**	**Mean**	**SD**	**SEM**
Confirmed leptospirosis	40	28.247	11.425	1.806
Severe leptospirosis	26	32.071	11.773	2.309
Mild leptospirosis	14	21.146	6.441	1.722
MAT equivocal	27	14.032	7.579	1.459
Non-leptospirosis fever	18	13.523	11.581	2.730
Healthy controls	23	6.078	2.802	0.572

### Comparison of NOx levels corrected for impaired renal function

**Figure 2:**
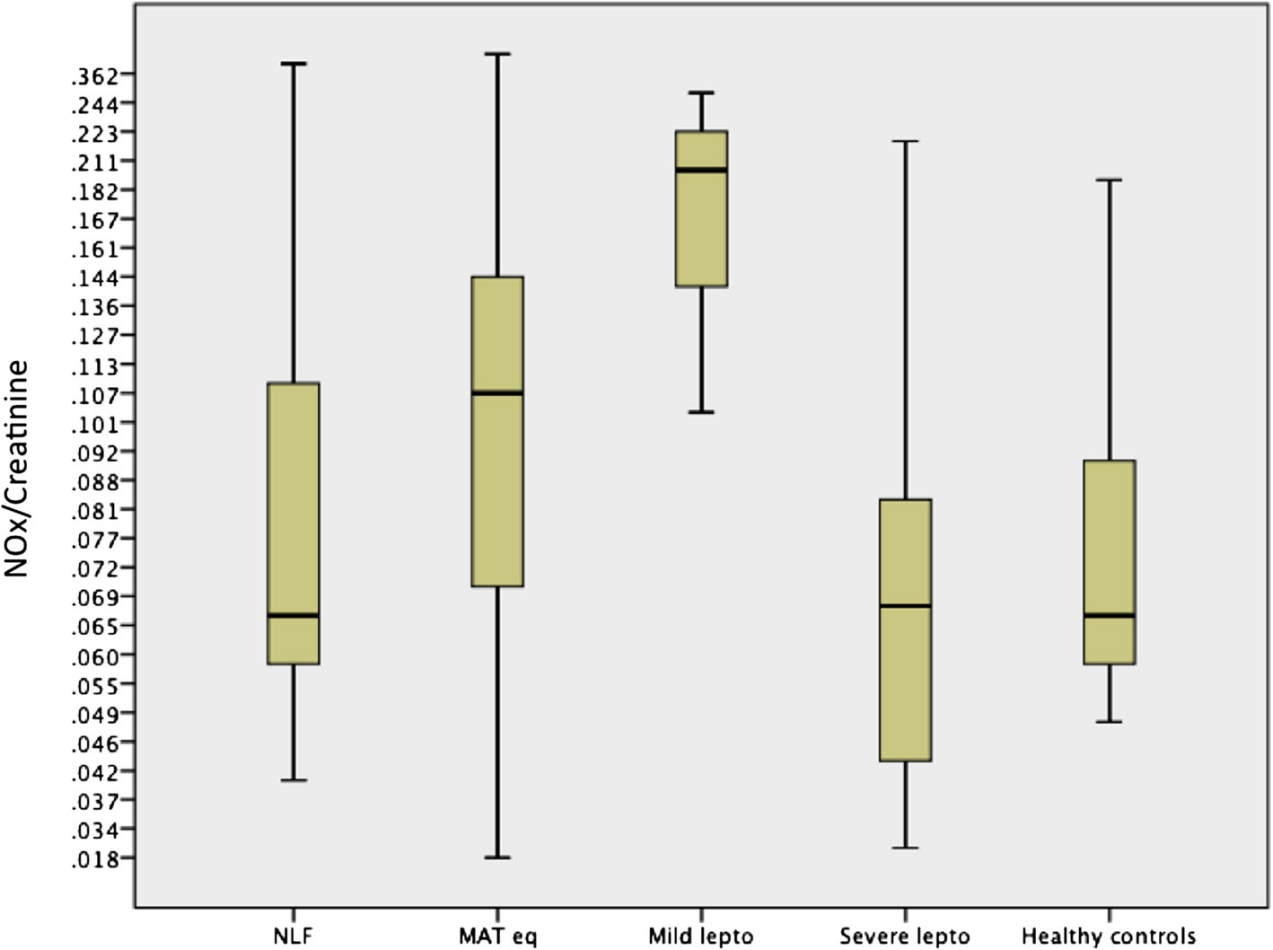
**Corrected NO**_**x **_**(NO**_**x**_**/creatinine) in different patient groups and healthy controls.** NLF: Non-leptospirosis fever; MAT Eq: MAT equivocal patients. lepto: leptospirosis.

In order to correct for renal impairment, we calculated the serum NO_x_/creatinine ratio in each of the groups. The comparisons of these groups are shown in Table 5 and Figure 2 (also Table 6). Corrected NO_x_ were lower among patients confirmed to have leptospirosis compared to healthy controls, although the difference was marginal. There was no significant difference seen in corrected NO_x_ levels among confirmed leptospirosis patients, MAT equivocal patients and NLFs.

**Table 5:** ANOVA post-hoc comparison using Bonferonni correction comparing NO_x_/creatinine in the different groups

**(I) Category**	**(J) Category**	**Mean difference (I-J)**	**Std. error**	**Sig.**
NLF	MAT eq	-.026593	.020710	1.000
	Mild Lepto	-.087484^*^	.024253	.005
	Severe lepto	.029598	.020869	1.000
	Controls	.019194	.021222	1.000
MAT eq	NLF	.026593	.020710	1.000
	Mild Lepto	-.060892	.022415	.077
	Severe lepto	.056191^*^	.018701	.033
	Controls	.045787	.019094	.183
Mild lepto	NLF	.087484^*^	.024253	.005
	MAT eq	.060892	.022415	.077
	Severe lepto	.117082^*^	.022562	.000
	Controls	.106679^*^	.022889	.000
Severe lepto	NLF	-.029598	.020869	1.000
	MAT eq	-.056191^*^	.018701	.033
	Mild Lepto	-.117082^*^	.022562	.000
	Controls	-.010404	.019266	1.000
Controls	NLF	-.019194	.021222	1.000
	MAT eq	-.045787	.019094	.183
	Mild Lepto	-.106679^*^	.022889	.000
	Severe lepto	.010404	.019266	1.000

NLF: Non-leptospirosis fever, MAT eq: MAT equivocal patients, lepto: leptospirosis, Sig: significance. * denotes statistically significant differences.

**Table 6:** NO_x_/creatinine levels in the different study groups

**Category**	**N**	**Mean**	**SD**	**SEM**
Confirmed leptospirosis	40	0.113	0.074	0.012
Severe leptospirosis	26	0.072	0.042	0.008
Mild leptospirosis	14	0.189	0.058	0.015
MAT equivocal	27	0.128	0.096	0.018
Non-leptospirosis fever	18	0.101	0.085	0.020
Healthy controls	23	0.082	0.038	0.008

The most significant finding was that corrected NO_x_ levels were markedly lower among patients with severe leptospirosis compared with both mild leptospirosis and MAT-equivocal patients. On the other hand, no difference was seen in corrected NO_x_ levels in severe leptospirosis patients, healthy controls and NLF patients. Corrected NO_x_ levels were significantly lower among healthy controls compared with mild leptospirosis patients and MAT equivocal patients, but no significant difference was shown between healthy controls and NLFs.

## Discussion

Elevation of serum NO_x_ levels during acute infections such as dengue, malaria and leptospirosis has been shown in previous studies, however the main criticism of these studies has been the lack of correction of NO_x_ concentrations for renal function. As mentioned above, NO_x_ is excreted predominantly by the kidneys, and NO_x_ levels have been shown to be elevated in the presence of renal impairment.

We demonstrated that crude NO_x_ levels are significantly elevated in leptospirosis, with higher levels correlating with severity of the illness. However, once NO_x_ levels were corrected for renal function, they were significantly lower in severe leptospirosis. If NO_x_ levels reflect iNOS activity, this finding suggests that iNOS activity is diminished in patients developing severe disease. Furthermore, the results suggest that iNOS activity is similar in severe leptospirosis and healthy controls despite the heavy inflammatory response in the former group. We postulate that this indicates that a blunted iNOS response is seen in severe leptospirosis; whether this is the result of the inflammatory response that occurs, or whether a diminished iNOS response plays a role in the genesis of severe leptospirosis and organ dysfunction remains to be elucidated. Conversely, it is possible that higher levels of NOx are protective against organ dysfunction. Admittedly, the relationship between iNOS activity and inflammation is extremely complex.

Nonetheless, similar patterns have been seen in malaria. Al Yaman et al. [10] described an association between high levels of NO_x_ levels and coma in children with cerebral malaria. Similarly Kremsner et al. [9] showed that higher levels of plasma NO were seen in severe malaria; however they also demonstrated that higher levels of NO was associated with accelerated recovery. The criticism of both these studies was that crude NO levels were considered, and no correction was made for deranged renal function (serum creatinine levels in the patients were not provided), and that the elevated NO levels could simply be related to reduced excretion of NO due to impairment of renal function [13]. In fact, Anstey et al. [11] demonstrated that when NO_x_ levels were corrected for renal function, using the ratio NO_x_/Creatinine, NO_x_ levels showed an inverse relationship with the severity of malaria. Corrected NO_x_ levels were lowest in patients with severe disease, while higher levels were seen in controls as well as those with asymptomatic disease, suggesting that high NO_x_ levels may protect against severe malaria. Furthermore, in a mouse model, Gramaglia et al. [26] demonstrated that low NO bioavailability contributes to the genesis of experimental cerebral malaria.

Although many confounding factors could be present, the finding that corrected NO_x_ levels are low in clinically severe leptospirosis is itself of significance. Whether NO_x_ levels are low in severe leptospirosis as a result of endothelial dysfunction resulting from severe disease, or whether individuals in whom iNOS activity does not increase in response to infection are more likely to develop severe disease remains unclear. Another possibility is that iNOS activity is normal, but the NO produced is rapidly removed by other molecules such as reactive oxygen species and haemoglobin. The measurement of serial NO_x_ levels and correlating these with the onset of organ dysfunction in patients with leptospirosis and in experimental models is likely to provide further insight into this issue.

The finding that corrected NO_x_ levels in NLFs were similar to those seen in severe leptospirosis is interesting. NLFs presumably represent a heterogenous group, and a significant number in this group had renal dysfunction. Whether this suggests a decrease in NO_x_ levels in infections which result in organ dysfunction is difficult to determine from this study. Clinical features similar to those of severe leptospirosis occur in many other infections, such as dengue, hanta-virus and acute hepatitis; some of the patients in the NLF and MAT equivocal groups did present with clinical features similar to severe leptospirosis; however numbers were too small for any realistic comparisons of NO levels in these subgroups to be possible.

The Griess reaction explained in the methodology is specific for nitrite levels in blood. Therefore in order to measure the nitrates, they had to be converted to nitrites. This conversion can be achieved with either a chemical or an enzymatic reduction and we opted for the chemical measure by treating the sera with Vanadium(III) chloride [7,14]. The use of Vanadium (III) chloride offers a low cost method compared to the enzymatic reduction and therefore the modified Griess assay is an inexpensive, simple, rapid, accurate and a sensitive method for measurement of NO_X_ levels, better suited method for resource limited settings in developing countries.

### Limitations

One of the limitations of this study is the confounding factors that can affect serum NO_X_ levels such as age and diet [27]. However, the mean ages of the subjects in the different groups were comparable, and dietary influence was minimized by collecting samples in the early mornings prior to the intake of food. It was also possible that patients with severe disease had a lower dietary contribution of nitrites. This is very difficult to quantify in a clinical study, and no standard methods for quantification exist. However in our study, we compared the incidence and severity of symptoms such as nausea, vomiting and loss of appetite, and there was no difference in the incidence of these symptoms in severe disease compared with non-severe disease. All patients, even the sickest, were able to eat and drink. There was a gender bias in the sample with more males than females. However, this is a well established epidemiological fact in leptospirosis in Sri Lanka, as it is the males who engage more in outdoor activities such as farming which is a major occupational risk factor for leptospirosis [21]. Of the serological tests to diagnose leptospirosis, MAT is the preferred method, and it is also the test recommended by the epidemiology unit of the Ministry of Health in Sri Lanka [21]. However, MAT serology may be insensitive in early acute-phase specimens [28]. Moreover, patients with fulminant leptospirosis may die before seroconversion occurs. A four-fold rise in MAT titer would have helped to differentiate patients with true leptospirosis in the MAT equivocal group [22,29]. However, many patients did not return for follow up visits after discharge and we had to restrict the leptospirosis confirmed group to those with high MAT titers ≥400. We did not obtain serial NO_x_ measurements in patients (due to logistical difficulties and limited resources) which would have enabled us to predict the earliest point at which NO_X_ levels would start to differ in those with severe disease, thus establishing the approximate earliest point it would be useful as a predictive marker. Based on the results of this initial study a larger multi-centre study was designed by us, and is currently in progress.

## Conclusions

This study shows that crude serum NO_X_ levels were significantly elevated in Sri Lankan patients with leptospirosis compared to healthy controls and non-leptospirosis fever patients; however once NO_x_ levels were corrected for serum creatinine, the relationship between NO_x_ levels and disease severity was shown to be strikingly different. Corrected NO_x_ levels were significantly suppressed in patients with severe leptospirosis. Thus, NO_x_ levels in patients with leptospirosis may be useful to predict severe disease, i.e., the presence of low NO_x_ levels (after correction for renal function) in leptospirosis may predict the development or organ dysfunction. We also demonstrated that chemical conversion of nitrates to nitrite with Vanadium (III) chloride and measuring of NO_X_ levels (modified Griess reaction) is a relatively cheap assay technique that can be employed in resource limited settings.

## Abbreviations

NO: Nitric oxide; NOx: Total nitrites and nitrates; MAT: Microscopic agglutination titre; iNOS: Inducible nitric oxide synthatase; NLF: Non-leptospirosis fever; WHO: World Health Organisation; MODS: Multi-organ dysfunction syndrome; ELISA: Enzyme-linked immunosorbent assay; NHSL: National Hospital, Colombo, Sri Lanka.

## Competing interests

The authors declare that they have no competing interests.

## Authors’ contributions

SR, SMH, HJDS and TK developed the initial concept for the study. TK and TV collected clinical data and samples. TK and SMH performed the laboratory measurements. PS performed leptospirosis diagnostic serology. SR, SMH, TK and CR analysed the data. TK, CR, SMH and SR wrote the first draft. All authors read and approved the final manuscript.

## Authors’ information

TK(MSc) was an MSc Research Student, and SMH(PhD) is Senior Lecturer, at the Institute of Biochemistry, Molecular Biology, and Biotechnology (IBMBB). PS(MD) is Consultant Microbiologist at the Medical Research Institute, Colombo. HJDS(MD,FRCP,DPhil) is Senior Professor in the Dept of Medicine, University of Kelaniya. TV(MBBS) was Research Associate, CR(MBBS, MD) is Lecturer, and SR (MD,FRCP) is Professor in the Dept of Clinical Medicine, University of Colombo. SR(MD,FRCP), SMH(PhD) and HJDS(MD,FRCP,DPhil) are senior researchers currently supervising a series of studies on leptospirosis.

## Pre-publication history

The pre-publication history for this paper can be accessed here:

http://www.biomedcentral.com/1471-2334/13/206/prepub
